# Treatment of Recurrent IgA Nephropathy After Kidney Transplantation: Case Report and Comprehensive Literature Review

**DOI:** 10.1002/ccr3.71474

**Published:** 2025-11-21

**Authors:** Ahmad Matarneh, Omar Salameh, Monika Koirala, Amanda Karasinski, Gurwant Kaur, Naman Trivedi, Nasrollah Ghahramani, Vaqar Shah

**Affiliations:** ^1^ Department of Nephrology Milton S. Hershey Medical Center Hershey Pennsylvania USA; ^2^ University Health Truman Medical Center Kansas City Missouri USA; ^3^ UPMC Lititz Lititz Pennsylvania USA

**Keywords:** allograft dysfunction, kidney transplantation, recurrent IgA nephropathy, sparsentan, targeted‐release budesonide

## Abstract

Recurrent IgA nephropathy after kidney transplantation remains a major cause of graft dysfunction. This case highlights successful management with targeted‐release budesonide combined with sparsentan, underscoring the importance of early recognition and the promise of emerging therapies to preserve long‐term allograft function.

## 1 Introduction

IgA nephropathy (IgAN) accounts for 20%–40% of primary glomerular diseases leading to end‐stage kidney disease (ESKD) worldwide [1]. Kidney transplantation improves survival and quality of life compared to dialysis, but disease recurrence in the allograft remains a major concern, with rates ranging from 9% to 61% depending on follow‐up duration and biopsy protocol [1, 2, 3]. Clinically significant recurrence contributes to graft dysfunction and can lead to graft loss in up to 30% of affected patients [4].

The pathogenesis is driven by persistent systemic abnormalities in IgA regulation rather than alloimmune incompatibility. Overproduction of galactose‐deficient IgA1 (Gd‐IgA1) in gut‐associated lymphoid tissue leads to the formation of anti‐glycan autoantibodies and circulating immune complexes, which deposit in the mesangium and activate the alternative and lectin complement pathways [5, 6]. This immune dysregulation continues after transplantation, explaining the high recurrence risk.

## 2 Case History and Examination

A 57‐year‐old man with end‐stage renal disease secondary to long‐standing hypertension and IgA nephropathy underwent a deceased donor kidney transplant in 2006. His transplant was done because he had progression of his IgA nephropathy. Posttransplant, he maintained stable allograft function with a baseline serum creatinine between 1.5 and 1.9 mg/dL. His maintenance immunosuppression consisted of tacrolimus, mycophenolate mofetil, and low‐dose prednisone, and he had no prior history of recurrent glomerulonephritis or acute rejection.

In October 2021, during routine follow‐up, he was found to have new‐onset proteinuria of 3.08 g/24 h and microscopic hematuria with 10–19 red blood cells per high‐power field. Serum creatinine remained within the baseline range. He denied systemic features such as fever, rash, arthralgia, or infectious symptoms. Blood pressure was well controlled, and there was no edema or other abnormal findings on examination.

### 2.1 Differential Diagnosis

The appearance of new proteinuria and hematuria in a kidney transplant recipient raised concern for several possibilities. Recurrent or de novo glomerulonephritis was considered, particularly IgA nephropathy, focal segmental glomerulosclerosis, or membranous nephropathy. Chronic antibody‐mediated rejection was a key differential but was not supported by histology. Calcineurin inhibitor nephrotoxicity was considered but renal function remained stable and the biopsy did not suggest this. Infectious causes such as BK virus nephropathy or viral‐associated glomerulonephritis were also considered but systemic features and serologic evaluation did not support them.

### 2.2 Investigations and Treatment

Laboratory evaluation confirmed significant proteinuria and persistent microscopic hematuria with stable renal function. Serologic work‐up excluded autoimmune and infectious causes. A kidney allograft biopsy demonstrated mesangial proliferation with IgA‐dominant immune complex deposition, consistent with de novo IgA nephropathy. Chronic changes were mild, with limited interstitial fibrosis and tubular atrophy, and there was no evidence of acute cellular or antibody‐mediated rejection.

The patient was initially managed with supportive therapy including renin–angiotensin–aldosterone system blockade with an angiotensin receptor blocker and initiation of a sodium–glucose cotransporter‐2 inhibitor. As proteinuria persisted above 3 g/24 h, targeted‐release budesonide (Nefecon) was started, followed shortly thereafter by sparsentan, a dual endothelin type A and angiotensin II receptor antagonist. Background immunosuppression with tacrolimus, mycophenolate mofetil, and prednisone was continued unchanged.

### 2.3 Conclusion and Results (Outcome and Follow‐Up)

Within four months of initiating targeted therapy, urinary protein excretion declined to 0.25 g/24 h, microscopic hematuria resolved, and serum creatinine remained stable at 1.5–1.9 mg/dL. No adverse events were observed with either agent. At six months, repeat urinalysis and 24‐h urine collection confirmed sustained proteinuria reduction below 0.5 g/24 h.

At one‐year follow‐up, renal function remained stable with a serum creatinine of 1.6 mg/dL, proteinuria persisted at < 0.5 g/24 h, and hematuria did not recur. Blood pressure and metabolic parameters were well controlled, and no adverse effects related to budesonide or sparsentan were documented. The patient continues under regular transplant clinic follow‐up with stable allograft function.

## 3 Discussion

Recurrent IgAN is detected in 20%–60% of kidney transplant recipients transplanted for IgAN [1, 2, 3]. The risk is influenced by follow‐up duration and whether surveillance (protocol) or indication biopsies are performed. Clinical presentation varies from subclinical mesangial IgA deposits detected incidentally to progressive proteinuria with gradual eGFR decline, or aggressive crescentic disease leading to rapid graft failure [4, 7].

Reported risk factors include:

Younger recipient age (< 40 years) [1, 8].

Rapid progression to ESKD in the native disease [8].

Living donor transplantation (possibly due to longer graft survival) [9].

Persistent posttransplant proteinuria (> 1 g/day) or microscopic hematuria [4, 10].

Early corticosteroid withdrawal or reduction in calcineurin inhibitor exposure [11, 12].

### 3.1 Pathophysiology

The “multihit” hypothesis for IgAN pathogenesis applies equally to recurrence:

1. Increased production of Gd‐IgA1 by mucosal B cells in gut‐associated lymphoid tissue.
2. Autoantibody formation against Gd‐IgA1.
3. Circulating immune complex formation and mesangial deposition.
4. Complement activation (alternative and lectin pathways), driving inflammation, mesangial proliferation, and fibrosis [5, 6].

The deposition of galactose‐deficient IgA1–containing immune complexes in the mesangium triggers activation of mesangial cells, leading to proliferation, cytokine release, and complement activation. This inflammatory cascade causes secondary podocyte injury and disruption of the glomerular filtration barrier, resulting in increased glomerular permeability to albumin and other plasma proteins [7]. The degree of proteinuria therefore reflects the extent of podocyte and mesangial injury rather than simply immune complex load [8]. In both native and recurrent IgA nephropathy, proteinuria serves as a key surrogate marker of disease activity and treatment response, allowing longitudinal monitoring without the need for repeated biopsies. Sustained reduction in proteinuria correlates strongly with improved long‐term allograft survival and reduced progression to chronic graft dysfunction.

Histologic recurrence is often characterized by mesangial hypercellularity, segmental sclerosis, endocapillary proliferation, and C3 deposition [7]. Aggressive forms may feature crescents and extensive complement activation.

### 3.2 Management Principles

#### 3.2.1 Supportive Therapy

Renin–angiotensin system (RAAS) blockade remains the foundation for proteinuric disease, supported by both transplant and native IgAN data [7, 13]. Blood pressure control (< 130/80 mmHg when tolerated) and dietary sodium restriction are standard.

SGLT2 inhibitors have shown benefit in reducing proteinuria and slowing CKD progression in native IgAN [14, 15], but transplant data are limited; use should be individualized with close monitoring for hemodynamic effects and infections.

#### 3.2.2 Immunosuppression Strategy

Maintenance corticosteroids may reduce recurrence risk. Multiple registry analyses (including UNOS/OPTN) link early steroid withdrawal to higher recurrence rates and poorer outcomes [11, 12]. If feasible, avoidance of premature steroid discontinuation is advised in recipients transplanted for IgAN.

Adjustments to calcineurin inhibitor dosing or antiproliferative therapy have not shown consistent benefit in reversing established recurrence; optimization of baseline immunosuppression and ensuring adherence remain essential. A summary of management strategies that have been reported in the literature is presented in Table 1.

**TABLE 1:** Reported therapies for recurrent IgA nephropathy after kidney transplant.

Therapy	Mechanism	Evidence in recurrence	Typical scenario
RAAS blockade	↓ Intraglomerular pressure, ↓ proteinuria	Standard of care [7, 13]	Any proteinuric recurrence
Maintain corticosteroids	Anti‐inflammatory, immunosuppressive	Registry/observational evidence [11, 12]	High‐risk IgAN recipients
TR‐budesonide	Local steroid at GALT → ↓ Gd‐IgA1	Native IgAN RCT [4, 16]; transplant case series [17, 18]	Persistent proteinuria
Sparsentan	Endothelin A & AT1 receptor blockade	Native IgAN RCT [19]; transplant case reports [20]	RAAS‐resistant proteinuria
Cyclophosphamide + steroids	Immunosuppression for proliferative lesions	Case reports [21]	Crescentic recurrence
Plasma exchange	Removes immune complexes	Case reports [22]	Rapidly progressive recurrence
Complement inhibitors	Block lectin/alternative complement pathway	Native IgAN trials [6, 23]; transplant case reports [22]	Complement‐rich, refractory recurrence
Rituximab	Anti‐CD20 B‐cell depletion	Small series [24]	Refractory immune‐complex activity
Tonsillectomy + steroids	Remove mucosal IgA source	Japanese series [25]	Select centers/patients
SGLT2 inhibitors	↓ Proteinuria, slows CKD	Native IgAN RCTs [14, 15]	Individualized use in KT

### 3.3 Targeted and Adjunctive Therapies Reported in Recurrence

#### 3.3.1 Targeted‐Release Budesonide (TR‐Budesonide)

This gut‐targeted corticosteroid delivers drug to Peyer's patches to suppress mucosal Gd‐IgA1 production. In the phase 3 NefIgArd trial in native IgAN, 9 months of TR‐budesonide reduced proteinuria by ~27% and slowed eGFR decline by ~50% compared to placebo over 2 years [4, 16]. In the transplant setting, case reports and small series document proteinuria reduction and stable graft function in recurrent IgAN, with acceptable tolerability [17, 18].

#### 3.3.2 Sparsentan

A dual endothelin type A and angiotensin II receptor blocker, sparsentan achieved greater and sustained proteinuria reduction than irbesartan in the phase 3 PROTECT trial in native IgAN [19]. Transplant experience is limited to case‐level reports showing feasibility and proteinuria improvement [20].

#### 3.3.3 Complement Pathway Inhibitors

Iptacopan (factor B inhibitor) improved proteinuria and eGFR slope in native IgAN in the APPLAUSE‐IgAN trial [6].

Narsoplimab (anti‐MASP‐2) demonstrated proteinuria reduction in early IgAN studies [23].

Eculizumab has been used successfully in isolated cases of crescentic recurrent IgAN refractory to conventional therapy [22]. Evidence in transplant recipients is limited to case reports, but complement inhibition may be considered in biopsy‐proven complement‐rich, aggressive recurrence.

#### 3.3.4 Other Reported Interventions

- Cyclophosphamide plus pulse corticosteroids: Case series report remission in crescentic recurrence [21].
- Rituximab: Mixed efficacy in small transplant series; reserved for refractory immune‐complex activity [24].
- Tonsillectomy plus steroid pulses: Used in Japanese centers with improvement in proteinuria/histology; not widely adopted internationally [25].
- Therapeutic plasma exchange: Applied in aggressive crescentic recurrence, often with steroids ± cyclophosphamide or complement blockade [22].

The role of plasma exchange in recurrent IgA nephropathy after kidney transplantation remains uncertain and is not supported by controlled studies. While plasmapheresis has shown some benefit in native crescentic IgAN by reducing circulating IgA–IgG immune complexes and improving renal outcomes in small case series, evidence in the transplant setting is anecdotal [22]. Reported use has been limited to aggressive crescentic or rapidly progressive cases, often combined with corticosteroids and cyclophosphamide or complement inhibition. Its mechanism is presumed to involve the removal of pathogenic immune complexes rather than alloantibody clearance, since recurrent IgAN is driven by persistent mucosal immune dysregulation rather than donor‐specific antibodies [24]. Given the lack of standardized protocols and limited transplant‐specific evidence, plasmapheresis should be considered only on a case‐by‐case basis, ideally in severe crescentic recurrence unresponsive to conventional therapy.

A summary of proposed treatments and reported therapies literature review is shown in Table 1.

## 4 Conclusion

Recurrent IgAN is a frequent and clinically important complication after kidney transplantation. It reflects persistent mucosal immune dysregulation and can present in indolent or aggressive forms. Management starts with optimized supportive therapy, careful maintenance immunosuppression, and early detection via urinalysis and biopsy.

Mechanism‐based treatments such as TR‐budesonide and sparsentan have strong evidence in native IgAN and growing case‐based support in transplant recipients. Complement inhibitors and other targeted approaches offer promise for aggressive or refractory disease. Until robust transplant‐specific randomized trials are available, therapy should be individualized, guided by histology, proteinuria burden, and patient comorbidities, with consideration of enrollment in clinical studies.

## Author Contributions

**Ahmad Matarneh:** conceptualization, writing – original draft, writing – review and editing. **Omar Salameh:** writing – original draft. **Monika Koirala:** writing – original draft. **Amanda Karasinski:** writing – original draft. **Gurwant Kaur:** writing – original draft. **Naman Trivedi:** writing – original draft. **Nasrollah Ghahramani:** conceptualization, supervision, writing – original draft, writing – review and editing. **Vaqar Shah:** writing – original draft.

## Ethics Statement

Ethical approval for this study was waived by the Penn State Health Ethical Committee because this is a case report.

## Consent

Written informed consent was obtained from the patient for publication of this case report and any accompanying images. A copy of the written consent is available for review by the Editor‐in‐Chief of this journal.

## Conflicts of Interest

The authors declare no conflicts of interest.

## Data Availability

The data that support the findings of this case report are available within the article. Additional anonymized information may be obtained from the corresponding author upon reasonable request, in accordance with patient privacy regulations and institutional policies.
