# Correction to “Treatment of Recurrent IgA Nephropathy After Kidney Transplantation: Case Report and Comprehensive Literature Review”

**DOI:** 10.1002/ccr3.71934

**Published:** 2026-01-26

A. Matarneh, O. Salameh, M. Koirala, et al., “Treatment of Recurrent IgA Nephropathy After Kidney Transplantation: Case Report and Comprehensive Literature Review,” *Clinical Case Reports* 13, no. 11 (2025): e71474, https://doi.org/10.1002/ccr3.71474.

In the published version of this article, errors were identified in the reference list limited to bibliographic citation details, including incorrect or incomplete author names, journal titles, publication years, volume or issue numbers, and page ranges. During the correction process, the reference list was reviewed against the original source publications and corrected where necessary to ensure accuracy and consistency with the cited literature. No references were added or removed, and no changes were made to the in‐text citations, scientific content, data interpretation, or conclusions of the article. The authors apologize for these errors.

Below are references that need to be corrected, mistakes that have been noted are pertaining to DOI's linking and names.

Reference #6

In Published version

C. Jäger, C. S. Wang, T. Keller, et al., “Recurrence of IgA Nephropathy After Kidney Transplantation,” *BMC Nephrology* **23** (2022): 164, https://doi.org/10.1186/s12882‐022‐02802‐x.

PubMed link incorrect; other links are correct.

Correct PubMed:

https://pubmed.ncbi.nlm.nih.gov/35538438/

Jäger C, Stampf S, Molyneux K, Barratt J, Golshayan D, Hadaya K, Huynh‐Do U, Binet FI, Mueller TF, Koller M, Kim MJ. Recurrence of IgA nephropathy after kidney transplantation: experience from the Swiss transplant cohort study. *BMC nephrology*. 2022 May 10; 23(1):178.

Reference #7

In Published version

N. Leeaphorn, N. Garg, E. V. Khankin, and F. Cardarelli, “Recurrence of IgA Nephropathy After Kidney Transplantation Among the US Population,” *Transplantation Direct* **3**, no. 4 (2017): e144, https://doi.org/10.1097/TXD.0000000000000642.

In new version

Leeaphorn N, Garg N, Khankin EV, Cardarelli F, Pavlakis M. Recurrence of IgA nephropathy after kidney transplantation in steroid continuation versus early steroid‐withdrawal regimens: a retrospective analysis of the UNOS/OPTN database. *Transplant International*. 2018 Feb; 31(2):175–86.

Reference #9

In Published version

T. Moriyama, K. Nitta, K. Suzuki, et al., “Latent IgA Deposition From Donor Kidney Is a Major Risk Factor for Recurrent IgA Nephropathy,” *Clinical Transplantation* **19**, no. Suppl 14 (2005): 41–47, https://doi.org/10.1111/j.1399‐0012.2005.00446.x.

DOI is incorrect.

In new version

The correct DOI

DOI: 10.1111/j.1399‐0012.2005.00403.x

Moriyama T, Nitta K, Suzuki K, Honda K, Horita S, Uchida K, Yumura W, Tanabe K, Toma H, Nihei H, Yamaguchi Y. Latent IgA deposition from donor kidney is the major risk factor for recurrent IgA nephropathy in renal transplantation. *Clinical transplantation*. 2005 Aug;19:41‐8.

Reference #10

In Published version

D. Maixnerová, A. Krajčovičová, J. Skibová, et al., “Outcome of 313 Czech Patients With IgA Nephropathy After Kidney Transplantation,” *Kidney International Reports* **6**, no. 9 (2021): 2315–2327, https://doi.org/10.1016/j.ekir.2021.06.024.

In new version

Maixnerova D, Hruba P, Neprasova M, Bednarova K, Slatinska J, Suchanek M, Kollar M, Novak J, Tesar V, Viklicky O. Outcome of 313 Czech patients with IgA nephropathy after renal transplantation. *Frontiers in Immunology*. 2021 Sep 30; 12:726215.

Reference #12

In Published version

J. Floege, “Recurrent IgA Nephropathy After Renal Transplantation,” *Seminars in Nephrology* **24**, no. 3 (2004): 287–291, https://doi.org/10.1016/j.semnephrol.2004.03.008.

In new version

The correct DOI

DOI: 10.1016/j.semnephrol.2004.01.008

Floege J. Recurrent IgA nephropathy after renal transplantation. In *Seminars in nephrology* 2004 May 1 (Vol. 24, No. 3, pp. 287‐291). WB Saunders.

Reference #18

In Published version

B. H. Rovin, H. J. L. Heerspink, J. Barratt, et al., “Efficacy and Safety of Sparsentan Versus Irbesartan in Patients With IgA Nephropathy (PROTECT): 2‐Year Results From a Randomised, Active‐Controlled, Phase 3 Trial,” *Lancet* **402**, no. 10416 (2023): 2034–2048, https://doi.org/10.1016/S0140‐6736(23)02088‐7.

In new version

DOI: 10.1016/S0140‐6736(23)02302‐4. Epub 2023 Nov 3

Rovin BH, Barratt J, Heerspink HJ, Alpers CE, Bieler S, Chae DW, Diva UA, Floege J, Gesualdo L, Inrig JK, Kohan DE. Efficacy and safety of sparsentan versus irbesartan in patients with IgA nephropathy (PROTECT): 2‐year results from a randomised, active‐controlled, phase 3 trial. *The Lancet*. 2023 Dec 2;402(10417):2077–90.

Reference #19

In Published version

H. J. L. Heerspink, V. Perkovic, R. M. Perkins, et al., “Early Proteinuria Reduction by Sparsentan Predicts Long‐Term Kidney Outcomes in IgA Nephropathy: Interim Results From PROTECT,” *Lancet* **401**, no. 10386 (2023): 95–106, https://doi.org/10.1016/S0140‐6736(22)02203‐3.

In new version

Heerspink HJ, Tesar V, Komers R, Hendry BM, Preciado P, Murphy E, Rovin BH. Implications of Proteinuria Remission on Estimated Glomerular Filtration Rate (eGFR) Trajectory in Patients with IgA Nephropathy in the PROTECT Trial: FR‐PO872. *Journal of the American Society of Nephrology*. 2024 Oct 1; 35(10S):10–681.

